# Transport target expression, a biomarker of brain shuttle fusion drug uptake, shows minimal influence of brain region, disease state, gender, and age, yet reveals significant individual variation

**DOI:** 10.1002/alz70855_103829

**Published:** 2025-12-24

**Authors:** Ana R. Santa‐Maria, Sanjid Shahriar

**Affiliations:** ^1^ Wyss Institute at Harvard University, Boston, MA, USA

## Abstract

**Background:**

Delivering drugs to the brain is challenging due to the blood‐brain barrier (BBB), which blocks over 99% of drugs, necessitating novel approaches for improved transport. We and others have developed brain shuttles that can be fused or conjugated to large molecule therapeutics to generate brain shuttle‐drug fusions with greatly enhanced brain uptake. Variation in expression levels of the transport receptor targets of these shuttle‐drug fusions is a critical variable in understanding variation in the clinical success of these new novel therapeutics. In this study, we have analyzed variation in expression of transport targets across brain region, diseases state, gender, age, and between individuals in each group.

**Method:**

To do this we have developed a bioinformatics pipeline to identify and quantify surface proteins on brain microvascular endothelial cells, utilizing publicly available single‐cell and single‐nucleus RNA sequencing (RNA‐Seq) datasets, as well as proteomic datasets from brain microvasculature. Our analyses targeted the expression of transport‐related proteins, in the context of brain sections from subjects with and without neuropathology. We conducted correlation analyses across multiple brain regions and disease states in Alzheimer's disease (AD). We examined variations in transport‐related protein expression with a focus on gender differences and aging, utilizing RNA‐Seq and proteomic data from individuals without cognitive impairment and those with AD.

**Result:**

Our findings reveal that factors such as brain region, disease state, gender, and aging do not significantly affect target transcript or protein expression, with consistent conclusions from both RNA‐Seq and proteomic analyses. However, we noted substantial individual variation within the same demographic cohorts in subjects with and without AD, suggesting that while demographic factors may not influence drug target expression, individual biological affect expression. Such differences in transport receptor expression levels may offer a biomarker to predict the uptake of drugs fused to shuttles targeting these receptors. Murine models in which transport receptor levels can be experimentally controlled would allow a more systematic investigation of these effects.

**Conclusion:**

Together, the current and planned studies can help to inform the development of personalized approaches to selecting patients for specific fusion drug treatments.

